# Investigation of an Active Focusing Planar Piezoelectric Ultrasonic Transducer

**DOI:** 10.3390/s24134082

**Published:** 2024-06-23

**Authors:** Qiao Wu, Bin You, Xu Zhang, Jun Tu

**Affiliations:** 1 School of Mechanical Engineering, Hubei University of Technology, Wuhan 430068, China; 2 Key Lab of Modern Manufacture Quality Engineering, Hubei University of Technology, Wuhan 430068, China

**Keywords:** focusing transducers, planar piezoelectric plates, FZP electrode patterns, silk screen printing technology

## Abstract

Ultrasonic focusing transducers have broad prospects in advanced ultrasonic non-destructive testing fields. However, conventional focusing methods that use acoustic concave lenses can disrupt the acoustic impedance matching condition, thereby adversely affecting the sensitivity of the transducers. In this paper, an active focusing planar ultrasonic transducer is designed and presented to achieve a focusing effect with a higher sensitivity. An electrode pattern consisting of multiple concentric rings is designed, which is inspired by the structure of Fresnel Zone Plates (FZP). The structural parameters are optimized using finite element simulation methods. A prototype of the transducer is manufactured with electrode patterns made of conductive silver paste using silk screen-printing technology. Conventional focusing transducers using an acoustic lens and an FZP baffle are also manufactured, and their focusing performances are comparatively tested. The experimental results show that our novel transducer has a focal length of 16 mm and a center frequency of 1.16 MHz, and that the sensitivity is improved by 23.3% compared with the conventional focusing transducers. This research provides a new approach for the design of focusing transducers.

## 1. Introduction

Ultrasonic focusing technology has a broad range of applications in industrial non-destructive testing [1,2,3,4], structural health monitoring [5,6], and medical ultrasound [7,8,9]. In recent years, high-quality research results in cutting-edge fields such as photo-acoustic imaging [10], ultrasonic neuromodulation [11], ultrahigh-frequency ultrasound [12], wireless power transfer [13,14], and acoustic tweezers [15] are reported continuously.

Conventional focusing transducers primarily use several passive focusing methods concerning acoustic concave lenses [16,17], Fresnel zone plates [18,19], or off-axis parabolic reflectors [20] to enhance the lateral resolution of transducers. However, these approaches have a significant problem, which is the loss of sensitivity of transducers [21]. Thickness-mode piezoelectric ultrasonic transducers are studied in this paper as an example. Such transducers typically consist of a piezoelectric plate, a matching layer, and a backing layer, with both the piezoelectric plate and the matching layer being planar thin plates. The ideal thickness of the matching layer should be 1/4 wavelength corresponding to the central frequency designed to ensure the maximum transmittance of the acoustic wave [22]. When an acoustic concave lens is attached to a planar transducer, the inhomogeneous thickness of the lens disrupts the thickness conditions of the acoustic impedance matching. Such an approach reduces the sensitivity of the transducer, resulting in a trade-off where the focusing transducer, despite converging the acoustic beam at the focal point and improving lateral resolution, sacrifices sensitivity. Other passive focusing methods, such as Fresnel zone plates and off-axis parabolic reflectors, introduce additional losses during the reflection and the propagation of acoustic waves. Curved piezoelectric structures [23], on the other hand, face challenges in adapting to optimal acoustic matching layers. Some studies develop acoustic metamaterials [24,25] or introduce phased array methods [26,27] to avoid these problems mentioned above. However, these solutions also present challenges in terms of fabrication complexity and cost-effectiveness.

This paper proposes a novel method for an active focusing planar piezoelectric ultrasonic transducer. The core of this method lies in the design and fabrication of multiple concentric ring electrodes in an FZP-like structure on a planar piezoelectric plate by utilizing screen-printing technology. Compared to conventional focusing methods, this method enhances the lateral resolution of the transducer while maintaining a high sensitivity. Additionally, this method offers the advantages of being easy to fabricate and inexpensive.

## 2. Fundamental Design of Active Focusing Planar Piezoelectric Plates

The conventional approach to focusing sound waves using a refractive lens involves adding a curved acoustic material in front of the piezoelectric element. The choice of material and its shape significantly affects the focusing effect and can disrupt the addition of an impedance matching layer. Additionally, parabolic reflectors are often bulky and challenging to set up. On the other hand, the Fresnel zone plate (FZP) is a diffractive device composed of alternating bright and dark zones, with decreasing radii towards the outer zones. The traditional design of a flat piezoelectric FZP focused transducer involves encapsulating the FZP structure as a zone plate at the front end of the probe, which is a passive focusing method that results in a partial loss of acoustic energy.

Therefore, by designing the electrodes on the planar piezoelectric plate according to FZP structure, active focusing of the planar piezoelectric plate can be achieved without the compromising of sensitivity, while also reducing insertion losses of the transducer. The original radius of the FZP is given by [21]

(1) $$r_{n}=\sqrt{\lambda Fn+\frac{\lambda^{2}n^{2}}{4}}$$

where *F* is the focal length, *λ* is the wavelength corresponding to the center frequency, *n* is the non-negative integer, and *r_n_* represents the radius of the nth zone of the Fresnel zone plate. Starting from the center, the first bright zone is a circular ring with a radius of *r*_1_, and the first dark zone extends from *r*_1_ to *r*_2_, and so on for *r_n_*. In this way, the diffracted sound waves pass through the alternating structure of bright and dark zones, ultimately leaving a bright focal spot at the focus.

The conventional principle of focusing in a traditional FZP transducer is based on the diffraction phenomenon that occurs when the plane wave signal generated by the transducer passes through the zone plate, which consists of alternating transparent and opaque regions. This diffraction process allows the plane wave to be focused at a specific location. Conventional FZPs have been widely used in the field of acoustics for focusing water-coupled or air-coupled transducers due to their excellent focusing performance. However, for transducer designs that prioritize sensitivity, such as air-coupled transducers, achieving this focusing effect at the cost of a direct loss in sensitivity (passive focusing) is not desirable.

**Figure 1:**
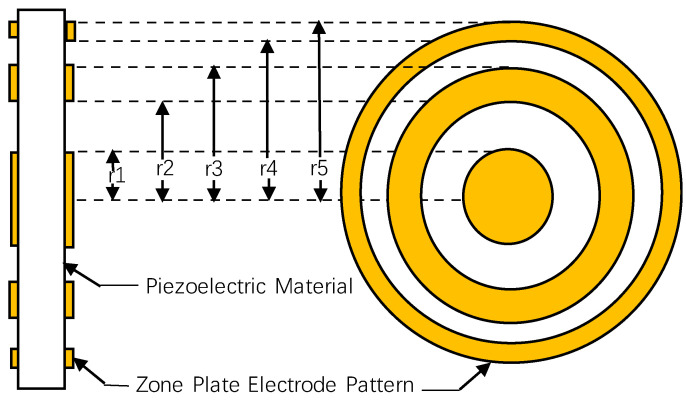
Multiple concentric ring electrode patterns: side view (**left**) and top view (**right**).

To achieve a focusing effect using multiple concentric ring electrodes similar to those of FZP + planar transducer, the radii of multiple concentric ring electrodes are initially determined based on the FZP structure, which is shown in Figure 1. Since electrical excitation signals can be applied where electrodes are present, the pattern of the multiple concentric ring electrodes is complementary to that of a conventional FZP. Electrodes are printed between the area where *r*_2*k*−1_ < *r* < *r*_2*k*_ (*r* is the radius, *k* = 1, 2, 3, …), with no electrodes in the remaining areas. The electrode pattern is implemented using screen-printing technology, which can achieve a precision of 0.1 mm, meeting the design requirements.

## 3. Finite Element Simulation and Optimization of the Electrode Pattern

To ensure that the multiple concentric ring electrodes in the FZP-like structure achieve the designed focusing effect, finite element simulation analysis [21] is conducted to optimize the radii parameters of each concentric ring. The entire piezoelectric plate in our design is continuous in radial direction, which means that even areas without electrode coverage can still generate some vibration, affecting the acoustic focusing. Therefore, unlike conventional passive focusing method using FZP structure design, it is necessary to optimize the radii parameters of the multiple concentric ring electrodes.

Referring to the FZP structure, the initial parameters for the multiple concentric ring electrodes are determined, as shown in Table 1. Using finite element simulation analysis, the acoustic pressure distribution along the transducer’s axial direction is obtained. The focal length and the radial sound pressure distribution at the focal point are then acquired. Based on the focusing characteristics of the transducer, including focal length, the peak-to-peak acoustic pressure value at the focal point, and the full-width at half-maximum (FWHM) in both the axial and radial directions, the radius parameters of the multiple concentric ring electrodes can be optimized. This process yielded the optimal structure parameters for the multiple concentric ring electrodes concerning some or all of the characteristics listed above.

**Figure 2:**
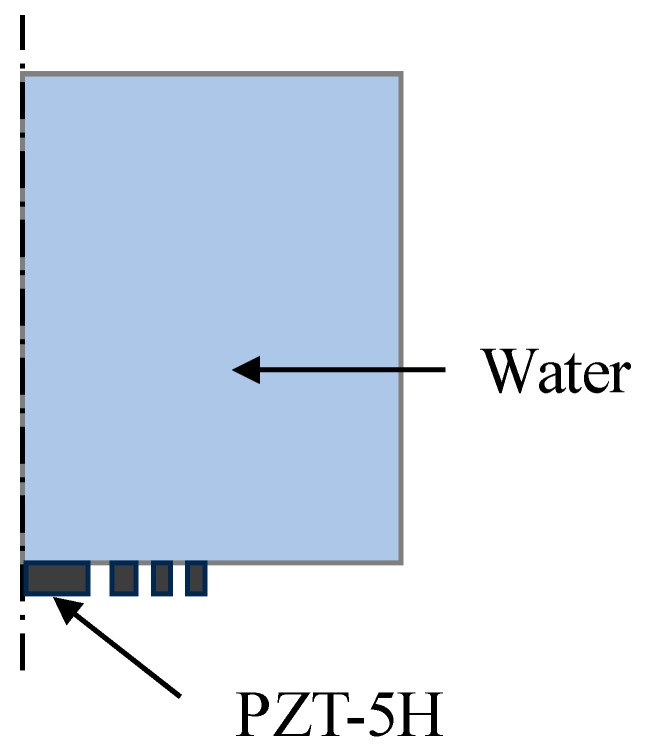
Simulation model for conventional passive focusing transducer.

**Figure 3:**
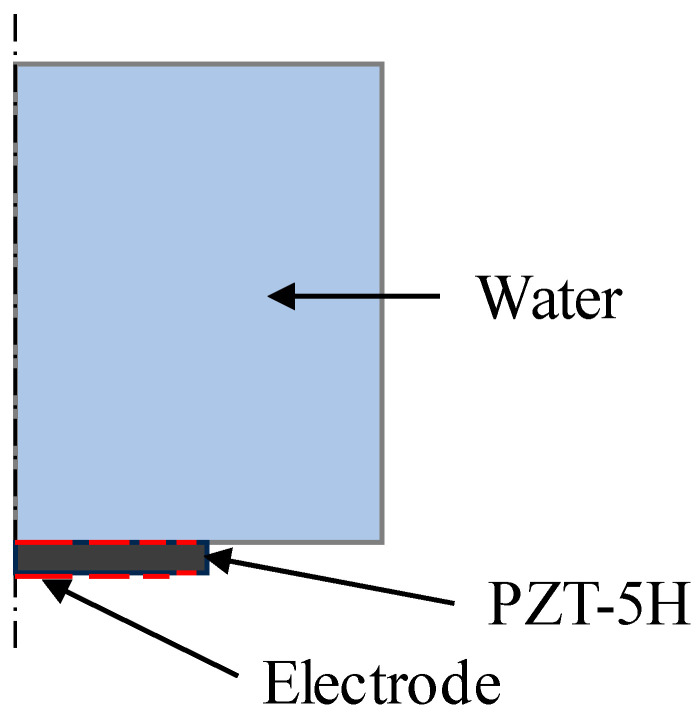
Simulation model for active focusing transducer using multiple concentric ring electrodes. The red dotted line indicates the multiple concentric ring electrodes.

Two-dimensional axisymmetric time-domain finite element simulation models are established, as shown in Figure 2 and Figure 3, to analyze both the conventional passive focusing transducer with an FZP baffle and the active focusing transducer based on the multiple concentric ring electrode structure proposed in our study. The simulations are conducted using COMSOL Multiphysics. In Figure 2 and Figure 3, the left boundary represents the axis of symmetry, while the top and right boundaries are absorbing boundaries. The transducer’s focal length is set to 15 mm, with a central frequency of 1 MHz. The piezoelectric material is PZT-5H with a diameter of 30 mm, and the medium is water. The mesh size is 1/15 of the minimum wavelength, and the signal of the electric source is a sinusoidal signal with a central frequency of 1 MHz.

In Figure 2, the piezoelectric plate is segmented into multiple non-contiguous concentric rings, representing the conventional passive focusing transducer using planar transducer with a FZP baffle. In Figure 3, the piezoelectric material is set as an entire thin plate, with electrodes arranged as multiple concentric rings according to the structure shown in Figure 1, simulating the active focusing structure.

**Figure 4:**
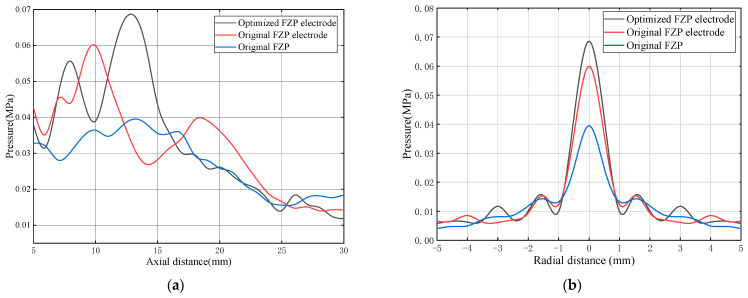
Simulation result of acoustic pressure: (**a**) axial and (**b**) radial. The blue, red, and black lines represent the planar piezoelectric plate with conventional FZP passive focusing, the active focusing planar transducer using the initial parameters, and the active focusing planar transducer using optimized parameters, respectively.

The simulation results are shown in Figure 4. Figure 4a,b shows the acoustic pressure distribution along the axial and radial directions, respectively. The blue, red, and black lines represent the planar piezoelectric plate with conventional FZP passive focusing, the active focusing planar transducer using the initial parameters, and the active focusing planar transducer using optimized parameters, respectively.

It can be seen from the simulation results, as expected, when the multiple concentric ring electrodes are determined using the initial FZP structure parameters, the continuity of the radial direction of the piezoelectric plate causes vibrations even in areas without electrode coverage, causing the transducer’s focal point to deviate from the originally designed position. However, the peak-to-peak acoustic pressure value at the focal point of active focusing transducer is much better, while the FWHM in both the axial and radial directions are at least as good as those of passive focusing transducers. Therefore, the minimum deviation of the transducer’s focal point and the maximum of the sensitivity are set as the joint optimization objective in this work, and are set to have equal weight. During the simulation process, it is discovered that the focal point position is primarily influenced by *r*_1_ and *r*_2_. In order to simplify the optimization process, *r*_1_ and *r*_2_ are mainly adjusted, while other radii of concentric rings vary with *r*_1_ and *r*_2_ in accordance with the principle of gradually reducing the electrode widths referring to the FZP structure. One set of the optimized structural parameters for multiple concentric ring electrodes that meet the design requirements is shown in Table 2. Under this set of parameters, the sensitivity of the active focusing planar transducer increased by 73% compared to the conventional FZP passive focusing transducer, while the lateral resolution remained essentially the same. The focal length of the planar active focusing transducer is 13 mm, and the FWHM in radial direction is 0.6 mm.

A finite element simulation is given utilizing the optimized electrode design. Figure 4a displays the axial acoustic pressure at *r* = 0, which was calculated. With an acoustic pressure magnitude of 69 kPa, the focal length is 13 mm along the axial direction. This is in line with the focusing point position of the FZP baffle transducer. Figure 4b displays the radial acoustic pressure at the focal point with a FWHM of 0.6 mm.

In this section, the simulation calculations are used to establish the focal point position and axial FWHM for the FZP piezoelectric transducer’s focusing effect. A model for simulating the FZP electrode radius was developed using these data. When the optimized FZP electrode radius transducer simulation model was put side by side with the FZP piezoelectric transducer, it was discovered that the latter had a smaller radial FWHM and greater focal acoustic pressure. Notably, compared to the FZP piezoelectric transducer, the improved FZP electrode radius produced greater focusing performance. The annular electrode’s radius *r*_1_ had the greatest influence on the FZP transducer’s overall focusing performance, according to observations made during the electrode radius optimization procedure. Therefore, special attention should be given to *r*_1_ when modifying the electrode radius.

In this section, an example of the finite element simulation and parameter optimization process is presented primarily using the minimum deviation of the transducer’s focal point and the maximum of its sensitivity as the joint optimization objective. It should be pointed out that following a similar procedure, the optimization objective is not limited. Characteristics of the transducer, such as the focal length, the peak-to-peak sound pressure value at the focal point, the axial and radial FWHM, and their weighted combinations, can also serve as objective functions for optimization. Active focusing planar ultrasonic transducers with different focal lengths can similarly undergo simulation and optimization design following this approach.

## 4. Experimental Results and Discussion

**Figure 5:**
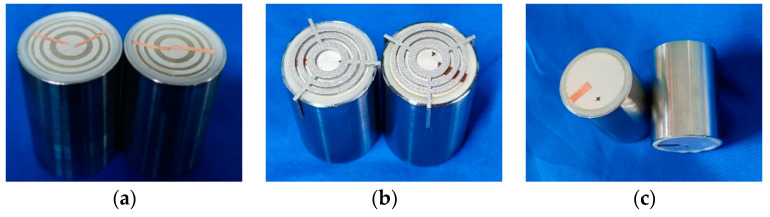
Different transducers fabricated in this work: (**a**) active focusing planar transducer, (**b**) conventional passive focusing transducer using FZP baffle, and (**c**) non-focusing transducer.

In this study, active focusing planar transducers using multiple concentric ring electrodes, conventional passive focusing transducers using an FZP baffle, and non-focusing transducers are fabricated, as shown in Figure 5a, Figure 5b, and Figure 5c, respectively. The diameter (30 mm), central frequency (1 MHz), and piezoelectric materials (PZT-5H) used for all three kinds of transducers are identical.

Based on the radii of the multiple concentric ring electrodes listed in Table 2, the parameters of the multiple concentric ring electrodes are given. Screen-printing technology is used in our work to fabricate multiple concentric ring electrode patterns with the material of conductive silver paste, and each electrode ring is connected with conductive tape. The width of the conductive tape is small enough, not affecting the acoustic field too much. A passive focusing transducer with a FZP baffle is also fabricated as a comparison. The baffle is fabricated utilizing 3D printing technology with an aluminum alloy substance.

The piezoelectric plate employed in the experiment needs to be housed in a 34-mm-diameter stainless-steel metal housing to move on to the following experimental tests. This shell protects the piezoelectric component from stray electromagnetic signals. A resin spacer must be added between the piezoelectric element and the metal housing to prevent a short circuit when the piezoelectric element is energized. The effective transducer diameter is 30 mm, and the piezoelectric element and metal housing will not interact with each other electrically. Figure 5a clearly illustrates the metal housing’s shape and how it corresponds to the FZP electrode piezoelectric transducer’s dimensions. In addition to providing mechanical protection, this encapsulating design guards against electrode corrosion and damage.

**Figure 6:**
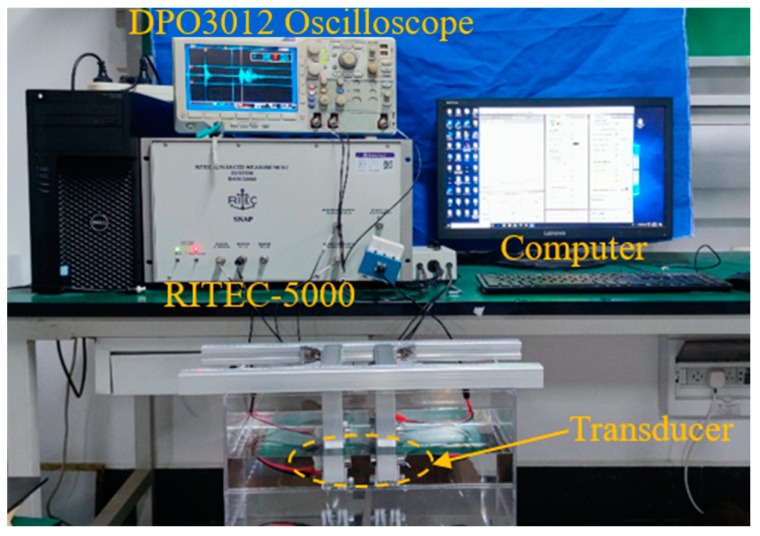
Transmitting receiving experimental platform.

As illustrated in Figure 6, an experimental test platform is set up to evaluate the acoustic characterizations of the active focusing transducers, passive focusing transducers and non-focusing transducers. To control the transducer’s axial and radial movements, the platform is mostly made of aluminum profiles and clamps. We can accurately position and monitor the transducer with the help of the ruler on the metal profiles. Testing the axial and radial acoustic field characteristics of the transducer is made easier by placing the complete device in a rectangular groove filled with silicone oil.

The transmission and receiving channels of the Ritec-5000 pulse generator are connected to the excitation probe and the receiving probe, respectively, in the experiment. A DPO3012 oscilloscope is used to display the output signal, and the working signal mode is set to one transmission and one receipt. The excitation source’s center frequency is set to 1 MHz by setting the excitation pulse count to 1 through a PC, matching the piezoelectric element’s center frequency. From 200 KHz to 2.5 MHz is the filter range.

**Figure 7:**
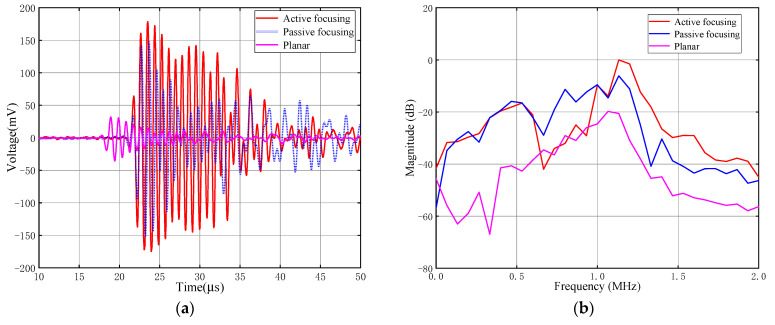
TR experimental results: (**a**) time-domain and (**b**) frequency spectrum. The red, blue, and pink lines represent the active focusing planar transducer using multiple concentric ring electrodes, the conventional passive focusing transducer using an FZP baffle, and the non-focusing transducer, respectively.

Transmitting receiving (TR) experiments of active focusing planar transducers using multiple concentric ring electrodes, conventional passive focusing transducers using an FZP baffle, and non-focusing transducers are carried out. Results are shown in Figure 7a, and Figure 7b, respectively. The active focusing, passive focusing, and non-focusing transducers are used as transmitting transducers, connecting to a Ritec-5000 device, which is set to generate a sinusoidal excitation signal. The electrical signal has a peak-to-peak voltage of 60 V and a central frequency of 1 MHz. A non-focusing ultrasonic planar transducer with the same central frequency is set as the receiving ultrasonic transducer, covered by a metal baffle plate having a central hole of 2 mm in diameter. Such avbaffle plate is fabricated by 3D printing technology using aluminum material, the acoustic impedance of which is large enough to block the acoustic waves. The main purpose of setting the receiving transducer in this way is to serve as a substitute for a hydrophone for subsequent acoustic field characterization. A hydrophone, of course, can be used as a receiving device here in the current experiments, but considering possible future experimental tests with air-coupled focusing ultrasonic transducers, we develop this testing approach instead.

Both transmitting and receiving transducers are aligned co-axially under identical experimental conditions. The time-domain receiving signals are shown in Figure 7a, and the frequency spectrum is shown in Figure 7b, with the spectrum normalized by the result of the active focusing transducer. The red, blue, and pink lines represent the active focusing planar transducer using multiple concentric ring electrodes, the conventional passive focusing transducer using an FZP baffle, and the non-focusing transducer, respectively. When the transmitting transducer is the non-focusing transducer, the receiving transducer is placed at a distance of 15 mm, which is also the designed focal length of the focusing transducers, being consistent with the focal length from previous finite element simulations.

As shown in Figure 7, the active focusing planar transducer using multiple concentric ring electrodes has a center frequency of 1.16 MHz and a −6 dB relative bandwidth of 10.3% in the frequency domain. The peak-to-peak value of the time-domain receiving signal is 371 mV, with a maximum spectrum value of −20.8 dB. The conventional passive focusing transducer using an FZP baffle has a center frequency of 1.14 MHz and a −6 dB bandwidth of 12.2%, with a time-domain receiving signal peak-to-peak value of 301 mV and a maximum spectrum value of −26.9 dB. The non-focusing transducer has a center frequency of 1.04 MHz and a −6 dB bandwidth of 23%, with a time-domain receiving signal peak-to-peak value of 68 mV and a maximum spectrum value of −40.6 dB.

Experimental results show that, compared to the conventional passive focusing transducer, the sensitivity of the active focusing transducer in the time domain is improved by 23.3%. In our work, since the electrical excitation signal and the receiving transducer are the same in each experiment, the sensitivity of the transducer can also be represented by the maximum value of the receiving signal spectrum in the TR system. In this case, the sensitivity is improved by 5.9 dB.

Compared to the conventional passive focusing transducer, the active focusing transducer developed in this study has a similar center frequency and −6 dB relative bandwidth under the same excitation conditions, but a significantly higher peak-to-peak value of both the time-domain and frequency-domain receiving signals at the focal point. This is mainly due to the active focusing method using multiple concentric ring electrodes based on an FZP-like structure, which avoids the energy loss caused by the reflection of the acoustic waves and other factors associated with the FZP baffle. Since the energy of the active focusing transducer is more concentrated around the center frequency, the sensitivity improvement is more significant when represented by the maximum value of the received signal spectrum. Because the frequency domain performance of the transducer is not the primary point of this study, we temporarily represent transducer sensitivity by the peak-to-peak value of the time-domain receiving signal, resulting in a sensitivity improvement of 23.3% for the active focusing transducer.

The active focusing planar piezoelectric transducer, passive focusing transducer, and non-focusing transducer are all tested. We measured the voltage signals from these three transducers in the axial direction over a distance of 10 to 30 mm, much like the simulations indicated before. With a step size of 1 mm, we also measured the voltage signals in the radial direction in the vicinity of the focus between 0 and 30 mm away.

**Figure 8:**
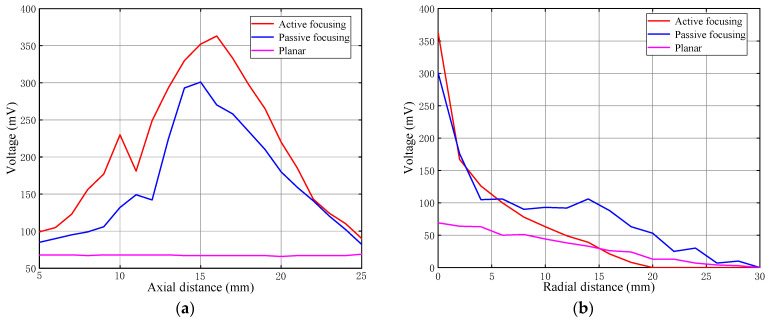
The experimental signals of the three transducers: (**a**) axial voltage signal and (**b**) radial voltage signal. The red, blue, and pink lines represent the active focusing planar transducer using multiple concentric ring electrodes, the conventional passive focusing transducer using an FZP baffle, and the non-focusing transducer, respectively.

Figure 8a shows the variation of axial sound pressure for the three transducers. It can be observed that the active focusing planar transducer achieves the highest voltage amplitude of 360 mV at the axial position of 16 mm, with a FWHM of 10.05 mm. In comparison, the conventional passive focusing transducer reaches a maximum voltage amplitude of only 300 mV at the axial position of 20 mm, with a FWHM of 9.40 mm. On the other hand, the non-focusing transducer exhibits relatively lower sound pressure amplitudes, averaging around 68 mV, and there is no significant focusing effect. Figure 8b presents the relationship between the radial distance from the focus and the voltage amplitude for these three transducers. The radial FWHM of the active focusing planar transducer is smaller, at 1.85 mm. On the other hand, the conventional passive focusing transducer has a slightly wider radial FWHM of 2.73 mm. The radial −6 dB width of the non-focusing transducer is 13.60 mm.

According to the examination of the experimental data presented above, the FZP electrode planar and FZP barrier planar piezoelectric transducers demonstrate significant axial focusing and have much narrower radial voltage widths than conventional transducers. These findings imply that FZP technology greatly enhances lateral resolution. Additionally, the traditional transducer generally exhibits poorer voltage amplitude performance. These results are extremely important for choosing and creating the best transducer for particular applications.

**Figure 9:**
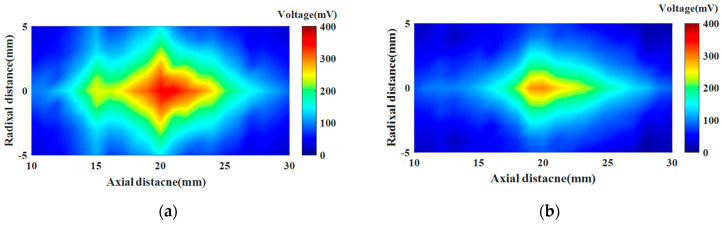
Acoustic field measurements of transducers with (**a**) active focusing and (**b**) passive focusing.

To further demonstrate the focusing effects of the FZP electrode transducer and FZP barrier transducer at the focal point, we conducted tests on the axial 10–30 mm electrical signals of these two transducers, as well as the radial −5–5 mm electrical signal distribution at each axial position. Figure 9a illustrates the acoustic field signals of the active focusing transducer in the axial 10–30 mm and radial −5–5 mm region, while Figure 9b represents the acoustic field signals of the passive focusing transducer in the same region. Comparison of the two figures shows that the FZP electrode-patterned transducer produces a brighter focal spot aggregated in this region, which produces a stronger energy of the sound pressure, and therefore, the energy utilization of this transducer is higher.

The acoustic field measurement results indicate that the active focusing planar transducer developed in this study has a focal length of 16 mm, which is close to the designed focal length. The discrepancy from the simulation results primarily arises from the precision errors inherent in the screen-printing process. During screen printing, conductive silver paste is applied on a 3D-printed mesh coating to create the multiple concentric ring electrode pattern. When the coating is removed, the silver paste may slightly flow outside the predetermined area, causing errors in the electrode pattern.

Additionally, in our study, a non-focusing receiving transducer covered by a metal baffle plate having a central hole of 2 mm in diameter is used instead of a hydrophone to obtain receiving signals, which may also introduce some error in the acoustic field measurement results. However, as previously mentioned, this acoustic field measurement method can be applied to the experimental testing of air-coupled ultrasonic transducers, where hydrophones cannot be used. Therefore, this method is still meaningful and the certain number of errors could be acceptable.

The active focusing planar ultrasonic transducer developed in this study exhibited a stronger sound pressure near the focal point. The focal length and the radial FWHM are similar to those of the conventional passive focusing transducer, while the axial FWHM is slightly increased, generally meeting the design expectations.

## 5. Conclusions

In this paper, a design method for active focusing planar piezoelectric ultrasonic transducers is introduced, centered on using screen-printing technology to create multiple concentric ring electrodes with a Fresnel zone plate (FZP)-like structure. Combining with acoustic matching layers of suitable thicknesses as an option, this method achieves beam focusing of planar acoustic waves while improving both the lateral resolution and sensitivity of the transducer. Comparing to conventional passive transducers, our novel focusing transducers has an improvement in sensitivity of 23.3%.

The design and optimization methods for the multiple concentric ring electrodes with an FZP-like structure are discussed in this paper. The initial structure parameters for the multiple concentric ring electrodes are derived directly from the FZP structure. Finite element simulation analysis is then used for the parameter optimization design, taking into account the differences between the multiple concentric ring electrode patterns and the conventional approach concerning a planar transducer with an FZP baffle. Factors such as the focal length, acoustic pressure level at the focal point, and the full width at half maximum (FWHM) of both the axial and radial directions are comprehensively considered during the optimization of the radii of the multiple concentric ring electrodes. Such an approach usually involves some trade-offs between the different characteristics of focusing transducers.

In this study, active focusing planar transducers using multiple concentric ring electrodes, conventional passive focusing transducers using an FZP baffle, and non-focusing transducers are fabricated, respectively. The designed diameter (30 mm), central frequency (1 MHz), and piezoelectric materials (PZT-5H) used for all three kinds of transducers are identical. The acoustic characteristics of the transducers above are experimentally tested. In the transmitting-receiving (TR) experiments using the same non-focusing receiving transducer covered by a metal baffle plate having a central hole of 2 mm in diameter, the results indicated that the active focusing planar ultrasonic transducer developed in this study achieved the maximum receiving signal at the focal point both in the time and the frequency domain. The radial FWHM is slightly narrower than that of the passive focusing transducer, while the axial FWHM is slightly larger. The experimental results show that our novel transducer has a focal length of 16 mm and a center frequency of 1.16 MHz, and that the sensitivity is improved by 23.3% compared with the conventional focusing transducers, validating the feasibility of the proposed design method. Our work in this paper provides a new approach for research on focusing piezoelectric ultrasonic transducers.

The more significant value of the proposed active focusing planar transducer design method lies in its potential application to the design of air-coupled focusing ultrasonic transducers. Air-coupled ultrasonic transducers, which do not utilize water, gel, or other liquid couplants, face a severe challenge on sensitivity due to the huge impedance mismatch at the solid–air interface. Conventional passive focusing methods used in focusing transducers, such as acoustic concave lenses, parabolic reflectors, and conventional FZP baffles, are thus less suitable for the design of air-coupled focusing transducers due to the sensitivity losses. Moreover, the acoustic impedance matching materials for air-coupled transducers usually have characteristics such as low acoustic velocity, thinness, brittleness, or softness, making it really challenging to fabricate curved acoustic matching layers with a uniform thickness. The focusing transducers with curved piezoelectric structures are thus also less suitable for the design of air-coupled focusing transducers.

The proposed design method for active focusing planar transducers, with its FZP-like multiple concentric ring electrodes patterns, avoids the sensitivity losses caused by acoustic wave reflection from conventional passive focusing transducers using FZP baffles. Such design also avoids the disruption of acoustic impedance matching thickness conditions caused by conventional acoustic concave lenses, and the difficulty of fabricating curved acoustic matching layers with a uniform thickness for curved piezoelectric structures. Thus, our design method for active focusing planar piezoelectric ultrasonic transducers holds significant promise especially for research on focusing air-coupled transducers. As a follow-up to this work, we will try to focus on the research of active focusing planar air-coupled transducers.

## Figures and Tables

**Table 1:** The radii of the multiple concentric ring electrodes.

number of rings	1	2	3	4	5	6	7
radius (mm)	4.8	6.9	8.5	10	11.3	12.5	13.6

**Table 2:** Optimized radii of the multiple concentric ring electrodes.

number of rings	1	2	3	4	5	6	7
radius (mm)	5.0	7.6	9.3	11.1	12.4	13.8	14.9

## Data Availability

The original contributions presented in the study are included in the article, further inquiries can be directed to the corresponding author.

## References

[1] Dong Z., Zhao P., Ji K., Chen Y., Gao S., Fu J. (2023). In-situ density measurement for plastic injection molding via ultrasonic technology. Front. Mech. Eng..

[2] Evani S.K., Spalvier A., Popovics J.S. (2021). Air-coupled ultrasonic assessment of concrete rail ties. NDT E Int..

[3] Fariñas L., Sanchez-Torres E.A., Sanchez-Jimenez V., Diaz R., Benedito J., Garcia-Perez J.V. (2021). Assessment of avocado textural changes during ripening by using contactless air-coupled ultrasound. J. Food Eng..

[4] Zhuo C., Zhao P., Ji K., Xie J., Ye S., Cheng P., Fu J. (2022). Ultrasonic measurement of tie-bar stress for die-casting machine. Front. Mech. Eng..

[5] Hsu D.K. (2006). Nondestructive testing using air-borne ultrasound. Ultrasonics.

[6] Wang Z., Hao D., Wang J., Zhang Y. (2022). Study on the acoustic field characteristics of OPCM focusing transducer. Mater. Today Commun..

[7] Philip N.S., Arulpragasam A.R. (2023). Reaching for the unreachable: Low intensity focused ultrasound for non-invasive deep brain stimulation. Neuropsychopharmacology.

[8] Bachu V.S., Kedda J., Suk I., Green J.J., Tyler B. (2021). High-intensity focused ultrasound: A review of mechanisms and clinical applications. Ann. Biomed. Eng..

[9] Yang Y., Yang M., Li C., Li R., Said Z., Ali H.M., Sharma S. (2023). Machinability of ultrasonic vibration-assisted micro-grinding in biological bone using nanolubricant. Front. Mech. Eng..

[10] Chen M., Jiang L., Cook C., Zeng Y., Vu T., Chen R., Lu G., Yang W., Hoffmann U., Zhou Q. (2022). High-speed wide-field photoacoustic microscopy using a cylindrically focused transparent high-frequency ultrasound transducer. Photoacoustics.

[11] Javid A., Ilham S., Kiani M. (2023). A Review of Ultrasound Neuromodulation Technologies. IEEE Trans. Biomed. Circuits Syst..

[12] Chen J., Fei C., Lin D., Gao P., Zhang J., Quan Y., Chen D., Li D., Yang Y. (2022). A review of ultrahigh frequency ultrasonic transducers. Front. Mater..

[13] Kim K., Jang S.G., Lim H.G., Kim H.H., Park S.M. (2021). Acoustic power transfer using self-focused transducers for miniaturized implantable neurostimulators. IEEE Access.

[14] Yi X., Zheng W., Cao H., Wang S., Feng X., Yang Z. (2021). Wireless power transmission for implantable medical devices using focused ultrasound and a miniaturized 1-3 piezoelectric composite receiving transducer. IEEE Trans. Ultrason. Ferroelectr. Freq. Control.

[15] Hou C., Li Z., Fei C., Zhao T., Sun X., Zhang J., Chen D., Yang Y. (2022). Composite ultrasound transducer for multi-size of tweezer manipulation. Appl. Acoust..

[16] Schwarz M., Eder D., Zagar B.G. (2020). Delay-and-sum processing of echo data of transducers focused by 3D printed lenses. Proceedings of the 2020 IEEE International Ultrasonics Symposium (IUS).

[17] Chen Z., Wei J., Song L., Song X. (2021). Optimal design of conical concave acoustic lens for large volumetric photoacoustic microscopy based on ray tracing. Proceedings of the Photons Plus Ultrasound: Imaging and Sensing 2021.

[18] Pan X., Zeng L., Li Y., Zhu X., Jin Y. (2023). Experimental demonstration of Fresnel zone plate lens for robust subwavelength focusing at mega hertz. Ultrasonics.

[19] Fuster J.M., Candelas P., Castiñeira-Ibáñez S., Pérez-López S., Rubio C. (2017). Analysis of fresnel zone plates focusing dependence on operating frequency. Sensors.

[20] Dai X., Zhu J., Tsai Y.T., Haberman M.R. (2011). Use of parabolic reflector to amplify in-air signals generated during impact-echo testing. J. Acoust. Soc. Am..

[21] Gomez Alvarez-Arenas T.E., Camacho J., Fritsch C. (2016). Passive focusing techniques for piezoelectric air-coupled ultrasonic transducers. Ultrasonics.

[22] Wu Q., Chen Q., Lian G., Wang X., Song X., Zhang X. (2021). Investigation of an air-coupled transducer with a closed-cell material matching strategy and an optimization design considering the electrical input impedance. Ultrasonics.

[23] He C., Wang Y., Lu Y., Liu Y., Wu B. (2016). Design and Fabrication of Air-Based 1-3 Piezoelectric Composite Transducer for Air-Coupled Ultrasonic Applications. J. Sens..

[24] Li Z., Zhao J., Hou C., Fei C., Zheng C., Lou L., Chen D., Li D., Yang Y. (2022). High-frequency self-focusing ultrasonic transducer with piezoelectric metamaterial. IEEE Electron. Device Lett..

[25] Hur S., Choi H., Yoon G.H., Kim N.W., Lee D.G., Kim Y.T. (2022). Planar ultrasonic transducer based on a metasurface piezoelectric ring array for subwavelength acoustic focusing in water. Sci. Rep..

[26] Ji W., Liu L., Xing Z., Zhang D., Wang Y., Chen L., Chen Y., Sun X., Du Y. (2020). Total-focus ultrasonic imaging of defects in solids using a PZT piezoelectric micromachined ultrasonic transducer array. IEEE Trans. Ultrason. Ferroelectr. Freq. Control.

[27] Shanmugam P., Iglesias L., Michaud J.F., Alquier D., Colin L., Dufour I., Certon D. (2021). Broad bandwidth air-coupled micromachined ultrasonic transducers for gas sensing. Ultrasonics.

